# Effective Elastic Modulus and Strengthening Mechanisms of CNT/Epoxy Composites: A Combined Theoretical and Experimental Study

**DOI:** 10.3390/ma19122650

**Published:** 2026-06-19

**Authors:** Yalei Wang, Jianqiu Zhou, Xiaohan Liu, Leilei Ding

**Affiliations:** 1 College of Mechanical Engineering, Nanjing Tech University, 30 Puzhu South Road, Nanjing 210000, China; wylwyl1990@126.com; 2 Nanjing Institute of Measurement and Testing Technology, 10 Xianlin Avenue, Nanjing 210000, China

**Keywords:** polymer composites, effective elastic modulus, interface strength, dynamic compression

## Abstract

Carbon nanotube (CNT)-reinforced composites are promising advanced materials due to their exceptional mechanical properties. This paper presents a comprehensive investigation of the mechanical behavior of CNT/epoxy composites through theoretical modeling and experimental validation. An equivalent cylindrical fiber model was developed to transform CNTs into effective reinforcement phases, enabling the application of classical composite mechanics. Three reinforcement configurations were analyzed: two unidirectional short fiber models (aligned and staggered) and a three-dimensional four-directional braided long-fiber model. The effects of geometric parameters, including the diameter-to-thickness ratio (D/t) and fiber aspect ratio, on the effective elastic moduli were systematically evaluated. Static and dynamic compression experiments were conducted using an MTS 810 testing system and a Split Hopkinson Pressure Bar (SHPB) to examine the influence of loading rate, vacuum treatment, and reinforcement type (CNT, SiC, and hybrid SiC/CNT) on composite strength. The results indicated that 3 wt% CNT reinforcement increases the Young’s modulus by 30% under static loading and enhanced the dynamic compressive strength under impact loading. The vacuum degassing process significantly affected composite quality, with insufficient vacuum leading to strength degradation due to void formation. Theoretical predictions using Mori–Tanaka and dilute methods showed good agreement with experimental results at low reinforcement volume fractions. Scanning electron microscopy revealed uniform CNT dispersion and provided insights into failure mechanisms, including CNT pull-out and breakage. This work contributes to the understanding of structure–property relationships in CNT-reinforced polymer composites and provides guidelines for achieving their optimal design.

## 1. Introduction

Since their discovery by Iijima in 1991, carbon nanotubes (CNTs) have attracted tremendous research interest owing to their extraordinary mechanical properties, including a Young’s modulus of up to 1 TPa and a tensile strength approaching 50 GPa [1]. These exceptional characteristics make CNTs ideal candidates for reinforcing high-performance composite materials. However, the effective incorporation of CNTs into polymer matrices faces several challenges, including achieving uniform dispersion, ensuring strong interfacial bonding, and developing reliable theoretical frameworks for property prediction.

The mechanical behavior of CNT-reinforced composites critically depends on load transfer efficiency between the CNT reinforcement and the polymer matrix. Unlike conventional fiber reinforcements with micrometer-scale diameters, CNTs possess nanoscale dimensions and extremely high aspect ratios, leading to unique interfacial phenomena [2]. The enormous specific surface area of CNTs (approximately 150 times greater than that of fibers with a diameter of 5 μm at an equivalent volume fraction) potentially enables more uniform stress distribution, yet it also introduces challenges related to agglomeration and poor wetting [3].

From a theoretical perspective, applying classical composite mechanics to CNT-reinforced systems requires appropriate homogenization techniques. Odegard et al. [4] proposed representative volume element (RVE) approaches that bridge molecular dynamics simulations with continuum mechanics. Hashin and Rosen [5] developed rigorous methods for fiber-reinforced composites that can be adapted for CNT systems. The equivalent fiber concept, wherein multi-walled or single-walled CNTs are transformed into equivalent solid cylindrical fibers with effective mechanical properties, provides a practical pathway for implementing micromechanical models.

The interface between CNTs and polymer matrices governs stress transfer efficiency and ultimately determines composite performance. Frankland et al. [6] demonstrated through molecular dynamics simulations that chemical cross-linking densities below 1% can enhance the interfacial shear strength by an order of magnitude compared with non-bonded interfaces. Lordi and Yao [7] identified polymer helical wrapping around CNTs as a key factor influencing interfacial friction and bonding. The critical aspect ratio required for achieving CNT fracture stress can be expressed as $L/D=\sigma_{max}/2\tau_{c}$, where $\tau_{c}$ represents the interfacial shear strength. More recent molecular dynamics studies have confirmed that modest functionalization or mechanical interlocking can elevate the interfacial shear strength to levels comparable to the matrix shear yield strength [8]. A comprehensive review of interfacial measurement techniques for CNT/polymer systems is given in [9].

Although several studies have investigated the compressive behavior of CNT/polymer composites [10,11], systematic research on the coupled effects of high loading rates and specific microstructures (e.g., three-dimensional braided architectures), as well as direct quantitative comparisons between theoretical models and dynamic experimental results, remains limited [12]. Furthermore, processing parameters such as vacuum degassing conditions significantly influence void content and, consequently, composite quality.

This study addresses these knowledge gaps through an integrated approach combining theoretical modeling with experimental validation. The objectives are to (1) develop equivalent continuum models for CNT-reinforced composites with different reinforcement architectures; (2) investigate the effects of geometric parameters (D/t, aspect ratio, and braiding angle) on the effective elastic properties; (3) conduct quasi-static and dynamic compression experiments to evaluate strengthening mechanisms; (4) examine the influence of processing conditions (vacuum level) on composite performance; and (5) compare theoretical predictions with experimental measurements.

## 2. Materials and Methods

### 2.1. Equivalent Cylindrical Fiber Model

**Figure 1:**
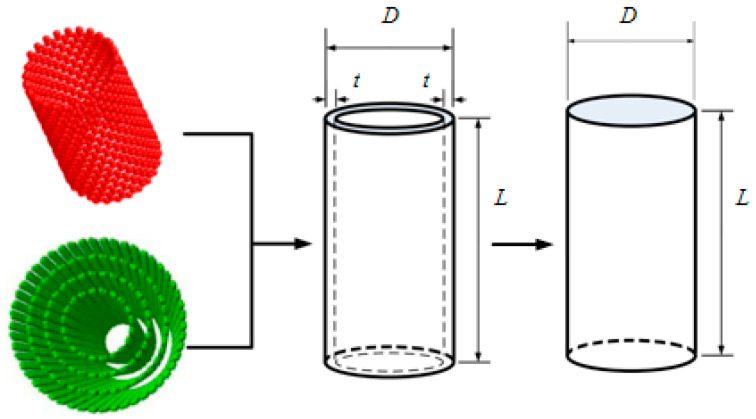
Equivalent process of carbon nanotubes.

The transformation of CNTs into equivalent cylindrical fibers follows the procedure illustrated in Figure 1. Single-walled or multi-walled CNTs are first idealized as cylindrical shells with an outer diameter D, wall thickness t, and length L. These shells are further homogenized into equivalent solid cylinders with identical outer diameter and length.

The equivalence condition requires that the axial stiffness of the CNT to be equal to that of the equivalent cylindrical fiber:

(1) $$E_{cnt}A_{cnt}=E_{cylindrical}A_{cylindrical},$$

where $E_{cnt}$ and $A_{cnt}$ represent the Young’s modulus and cross-sectional area of the CNT, respectively. The CNT cross-sectional area is given by

(2) $$A_{cnt}=\pi {\left(\frac{D}{2}\right)}^{2}-\pi {\left(\frac{D}{2}-t\right)}^{2}=\pi \left(D-t\right)t.$$

The equivalent Young’s modulus of the cylindrical fiber becomes

(3) $$E_{cylindrical}=\frac{4\left(D-t\right)t}{D^{2}}E_{cnt}=\frac{4\left(\frac{D}{t}-1\right)}{{\left(\frac{D}{t}\right)}^{2}}E_{cnt}.$$

**Figure 2:**
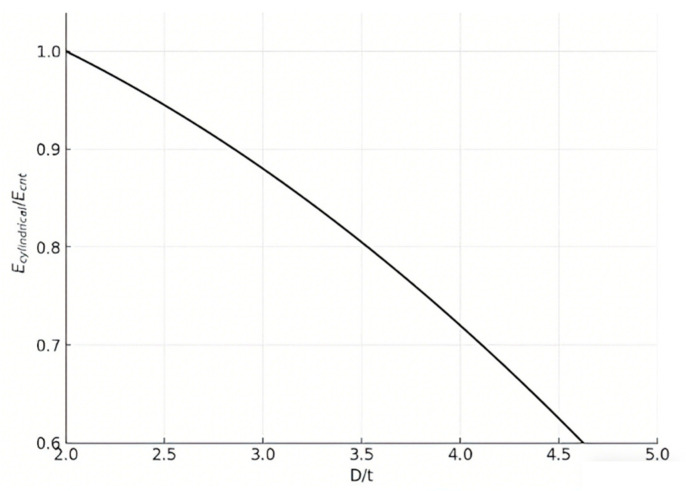
Relationship curve between the ratio of equivalent Young’s modulus to carbon nanotube Young’s modulus and D/t.

For D/t approaching 2, the CNT approaches a solid cylinder, and $E_{cylindrical}$ approaches $E_{cnt}$. As D/t increases, the equivalent modulus decreases rapidly, as shown in Figure 2.

The equivalent continuum treatment of CNTs as solid fibers has been widely adopted in recent multiscale frameworks, where the effective longitudinal modulus is extracted from atomistic or first-principles calculations and then incorporated into high-scale structural models [13].

It should be emphasized that this equivalent cylindrical fiber model serves as an engineering homogenization technique within the framework of classical micromechanics. It captures the dominant axial stiffness contribution of the hollow CNT while filtering out atomistic-scale details that are beyond the scope of a continuum treatment. Phenomena such as interfacial van der Waals slip, covalent cross-linking effects, and CNT waviness are not explicitly represented; these factors may become important for strength predictions or for highly non-linear deformation. As will be shown in Section 4.2, the predicted effective modulus at a practical volume fraction of 2 vol% is in close agreement with the experimental measurement, confirming that the idealization is adequate for elastic property estimation in well-dispersed systems.

### 2.2. Unidirectional Short Fiber Models

**Figure 3:**
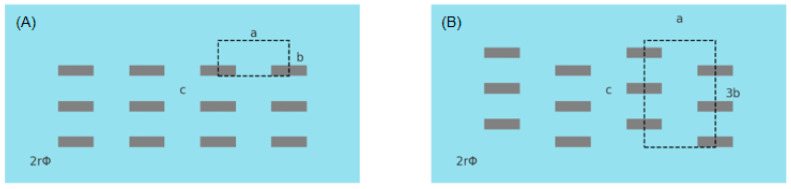
Schematic of (**A**) aligned and (**B**) staggered CNT arrangements in a representative volume element.

Two idealized configurations are considered: aligned CNT arrays and staggered CNT arrangements (Figure 3). The representative unit cell has length *a* and width *b*, with CNTs of length *c* and radius *r*. Assuming a uniform CNT distribution and satisfaction of minimum aspect ratio requirements, the longitudinal Young’s modulus under constant strain conditions is estimated using the simple rule of mixtures:

(4) $$E_{f}\;={\;E}_{\mathrm{lcnt}}V_{\mathrm{cnt}}+E_{m}V_{m},$$

where $E_{\mathrm{lcnt}}$ and $V_{\mathrm{cnt}}$ are the equivalent axial Young’s modulus and volume fraction of CNTs, and $E_{m}$ and $V_{m}$ are the corresponding matrix properties, with $V_{\mathrm{cnt}}$ + $V_{m}$ = 1. For identical CNT and matrix properties, both aligned and staggered configurations yield the same longitudinal modulus according to Equation (4). However, the staggered arrangement provide enhanced crack resistance by preventing crack propagation through CNT ends.

The transverse Young’s modulus under an isostress condition is estimated using the Reuss model (inverse rule of mixtures), which provides a lower bound:

(5) $$\frac{1}{E_{t}}=\frac{V_{\mathrm{cnt}}}{E_{\mathrm{tcnt}}}+\frac{V_{m}}{E_{m}},$$

where $E_{t}$ represents the equivalent transverse Young’s modulus of CNTs.

### 2.3. Three-Dimensional Four-Directional Braided Long Fiber Model

To address the anisotropy and delamination susceptibility of unidirectional composites, a three-dimensional (3D) four-directional braided configuration is considered. The local stiffness matrix [*Cf*] and compliance matrix [*Sf*] in the coordinate system (O^I^-123) are transformed into the global coordinate system using the transformation matrix [*Tk*]:

(6) $$\left[{\bar{C}}_{f}\right]=\left[T_{k}\right]\cdot \left[C_{f}\right]\cdot {\left[T_{k}\right]}^{T},$$

(7) $$\left[T_{k}\right]=\left[\begin{array}{cccccc} l_{1}^{2} & m_{1}^{2} & n_{1}^{2} & 2m_{1}n_{1} & 2m_{1}l_{1} & 2l_{1}m_{1}\\ l_{2}^{2} & m_{2}^{2} & n_{2}^{2} & 2m_{2}n_{2} & 2m_{2}l_{2} & 2l_{2}m_{2}\\ l_{3}^{2} & m_{3}^{2} & n_{3}^{2} & 2m_{3}n_{3} & 2m_{3}l_{3} & 2l_{3}m_{3}\\ l_{2}l_{3} & m_{2}m_{3} & n_{2}n_{3} & m_{2}n_{3}+m_{3}n_{2} & n_{2}l_{3}+n_{3}l_{2} & l_{2}m_{3}+l_{3}m_{2}\\ l_{3}l_{1} & m_{3}m_{1} & n_{3}n_{1} & m_{3}n_{1}+m_{1}n_{3} & n_{3}l_{1}+n_{1}l_{3} & l_{3}m_{1}+l_{1}m_{3}\\ l_{2} & m_{1}m_{2} & n_{1}n_{2} & m_{1}n_{2}+m_{2}n_{1} & n_{1}l_{2}+n_{2}l_{1} & l_{1}m_{2}+l_{2}m_{1} \end{array}\right],$$

(8) $$\left[C_{f}\right]=\left[\begin{array}{cccccc} c_{11} & c_{12} & c_{13} & 0 & 0 & 0\\ c_{12} & c_{11} & c_{13} & 0 & 0 & 0\\ c_{13} & c_{13} & c_{33} & 0 & 0 & 0\\ 0 & 0 & 0 & c_{44} & 0 & 0\\ 0 & 0 & 0 & 0 & c_{44} & 0\\ 0 & 0 & 0 & 0 & 0 & c_{66} \end{array}\right],$$

and

(9) $$\left[S_{f}\right]=\left[\begin{array}{cccccc} 1/E_{1} & -v_{12}/E_{1} & -v_{13}/E_{3} & 0 & 0 & 0\\ -v_{12}/E_{1} & 1/E_{1} & -v_{13}/E_{3} & 0 & 0 & 0\\ -v_{13}/E_{3} & -v_{13}/E_{3} & 1/E_{3} & 0 & 0 & 0\\ 0 & 0 & 0 & 1/G_{13} & 0 & 0\\ 0 & 0 & 0 & 0 & 1/G_{13} & 0\\ 0 & 0 & 0 & 0 & 0 & 1/G_{12} \end{array}\right].$$

The transformation matrix components *li*, *mi*, and *ni* (i = 1, 2, and 3) represent direction cosines between the local and global coordinate systems. For four-directional braiding with CNT orientations defined by angles (*θ*,*α*), the spatial orientations are CNT1: (θ,α), CNT 2: (θ,π-α), CNT 3: (π-θ,π-α), and CNT4: (π-θ,α).

The stiffness matrix of the carbon nanotubes in the overall coordinate system can be expressed as

(10) $$\left[C^{I}\right]=\frac{1}{4}\sum_{k=1}^{4}\left[T_{k}\right]\left[C_{f}\right]{\left[T_{k}\right]}^{T}.$$

The overall stiffness matrix of the three-dimensional four-way braided composite material obtained by filling the network structure woven from carbon nanotubes within the matrix is

(11) $$\left[C\right]=V_{f}\left[C^{I}\right]+V_{m}\left[C_{m}\right],$$

where $V_{f}$ is the volume fraction of carbon nanotubes and $V_{m}$ is the volume fraction of the matrix.

It should be noted that Equation (11) represents the Voigt (isostrain) approximation and thus provides an upper bound for the composite stiffness. For randomly oriented or partially aligned short CNTs, more rigorous homogenization schemes are required. The Mori–Tanaka method [14], which accounts for inclusion shape, orientation distribution, and inclusion–matrix interaction, is employed in Section 4.2 to compute the effective elastic modulus at experimentally relevant volume fractions. The Eshelby equivalent inclusion principle underlies the Mori–Tanaka estimate and yields results that lie between the Voigt and Reuss bounds.

## 3. Calculation Results and Analysis

Using material parameters reported in the literature [15] for CNTs with a diameter 1.38 nm ($E_{\mathrm{lcnt}}$ = 450.4 GPa and $E_{\mathrm{tcnt}}$ = 9.9 GPa) and epoxy matrix ($E_{m}$ = 7.2 GPa), the longitudinal and transverse Young’s moduli were computed for varying aspect ratios (c/a), where c is the CNT length and a is the RVE width. To systematically explore the influence of geometric parameters on the effective elastic moduli, a parametric study was conducted for CNT volume fractions ranging from 5% to 20%. It should be noted that such high volume fractions are rarely achieved in practice owing to processing constraints; the adopted range serves to reveal clear trends and the theoretical upper-limit performance of the composites. Our subsequent experiments were carried out at a CNT loading of 3 wt% (≈2 vol.%), which is representative of well-dispersed systems.

To establish a direct model–experiment connection, the effective elastic modulus was computed solely at the experimentally realized CNT volume fractions. The close agreement confirms that the equivalent-fiber homogenization framework is valid under practical processing conditions. To validate the proposed homogenization framework, the elastic modulus of the 3 wt% CNT composite was calculated at the experimentally relevant volume fraction of 2 vol%. The effective CNT modulus was taken as Ecylindrical = 135 GPa (obtained from Equation (3) for a typical D/t = 15). Applying the Mori–Tanaka method for randomly oriented, high-aspect-ratio inclusions [16] yields a predicted composite modulus of 2150 MPa, which is within 3.2% of the experimentally measured value of 2222 ± 58 MPa. The slight under-prediction can be attributed to a small fraction of aligned CNTs near the mold walls, as observed in SEM images. This agreement demonstrates that the equivalent-fiber model, combined with an appropriate homogenization scheme, captures the elastic behavior of well-dispersed CNT composites at realistic volume fractions.

**Figure 4:**
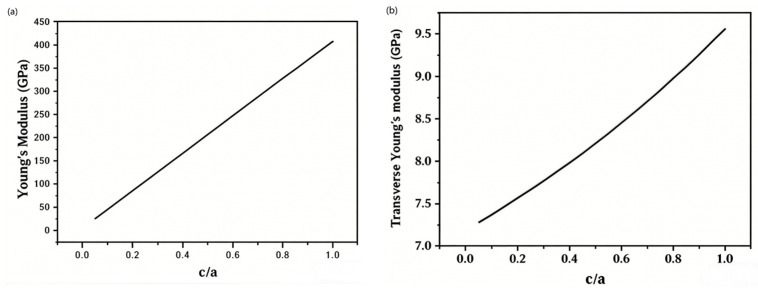
(**a**) Longitudinal Young’s modulus; (**b**) transverse Young’s modulus.

Figure 4a shows that the longitudinal Young’s modulus increases monotonically with c/a, approaching the continuous fiber limit at c/a = 1. For c/a = 0.5, $E_{t}$ ≈ 220 GPa, representing approximately 49% of the continuous fiber value. The transverse modulus (Figure 4b) exhibits a similar increasing trend but with a lower magnitude due to the relatively low transverse CNT modulus. These results demonstrate that maximizing the CNT aspect ratio is crucial for achieving optimal reinforcement efficiency. In practice, CNTs often exhibit aspect ratios exceeding 1000, which would theoretically yield properties close to continuous fiber composites. However, issues such as agglomeration, waviness, and imperfect alignment can reduce the effective aspect ratio, underscoring the importance of processing control.

For the 3D four-directional braided model, the material constants were selected as follows: $E_{1}$ = $E_{\mathrm{tcnt}}$ = 9.9 GPa, $E_{3}$ = $E_{\mathrm{tcnt}}$ = 450.4 GPa, $E_{m}$ = 7.2 GPa, G_12_ = 4.4 GPa, G_13_ = 27 GPa, $v_{12}$ = 0.16, $v_{13}$ = 0.3, and $v_{m}$ = 0.3, with a=π/4. Calculations were performed for CNT volume fractions $V_{f}$ = 35%, 40%, and 45%.

**Figure 5:**
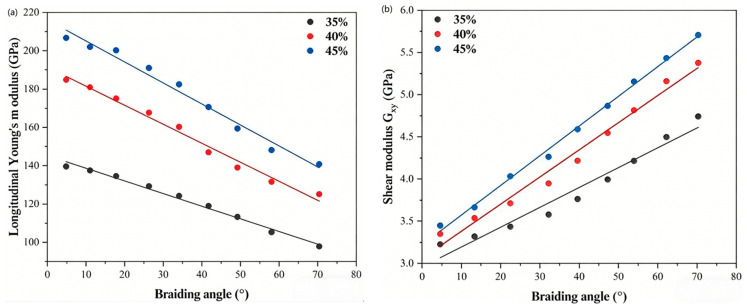
(**a**) Young’s moduli ($E_{x}$, $E_{y}$, and $E_{z}$) and (**b**) shear moduli (G_xy_, G_yz_, and G_zx_) as functions of the braiding angle θ for different CNT yarn volume fractions.

Figure 5a presents the variation of Young’s modulus with braiding angle θ. The longitudinal modulus $E_{z}$ decreases with increasing θ, while the transverse moduli $E_{x}$ and $E_{y}$ increase. This behavior reflects the progressive alignment of CNTs away from the longitudinal direction as θ increases. At θ = 0°, $E_{z}$ reaches its maximum value, approaching the rule-of-mixtures prediction, while at θ = 90°, $E_{z}$ minimizes but $E_{x}$ and $E_{y}$ maximize. The shear moduli (Figure 5b) show similar trends, with G_xy_ and G_yz_ increasing with θ. These results provide a design map for tailoring composite anisotropy: for applications requiring high axial stiffness, small braiding angles are preferred; for balanced multi-axial properties, intermediate angles (e.g., 30–45°) offer a compromise.

## 4. Experiments

Epoxy resin (E51, epoxy equivalent 184–200 g/eq) was used as the matrix material, with methyl tetrahydrophthalic anhydride (MeTHPA) as the curing agent and 2,4,6-tris(dimethylaminomethyl)phenol (DMP-30) as the accelerator. The resin-to-curing-agent mass ratio was 100:80, with 1 phr (parts per hundred resin) of DMP-30 accelerator, consistent with the manufacturer’s recommended formulation for E51/MeTHPA systems. The materials were first mechanically mixed. Multi-walled carbon nanotubes (diameter 20–40 nm, length 10–30 μm, purity > 95%, supplied by NIST, Gaithersburg, MD, USA) and silicon carbide particles (diameter 4 μm, supplied by Sigma-Aldrich, St. Louis, MO, USA) were used as reinforcements. The materials were first mechanically mixed using a high-shear mixer at 2000 rpm for 30 min, followed by ultrasonication (20 kHz, 50% amplitude, 15 min) to achieve uniform dispersion. Composites with different reinforcement contents were fabricated: CNT at 0.2, 0.4, 0.8, 1.2, and 3.0 wt%; SiC at 0.8 and 1.2 wt%; and a hybrid composition containing 3 wt% SiC with CNTs. The materials were mixed mechanically, degassed under either low vacuum (approximately 0.1 MPa) or high vacuum (0.01 MPa) to investigate the effect of void removal, and then cast into cylindrical molds. All specimens were cured at room temperature for 24 h, followed by post-curing at 80 °C for 4 h. Final specimens were machined to dimensions of 10 mm in diameter and 10 mm in length for compression testing.

We used a universal material testing machine, a Hopkinson pressure bar impact standard device, and a scanning electron microscope to test and characterize the above samples. The mechanical testing equipment consisted of a universal testing machine (MTS 810, MTS Systems Corp., Eden Prairie, MN, USA) for quasi-static tests, a split Hopkinson pressure bar (SHPB) apparatus for dynamic tests, and a scanning electron microscope (SEM, ZEISSEVO10, Oberkochen, Germany) for fractography.

### 4.1. Experimental Conditions

Quasi-static compression tests were performed on an MTS 810 servo-hydraulic testing machine. In accordance with ASTM D695-15, with the specimen dimensions modified to φ 10 mm × 10 mm (diameter × height) owing to mold constraints. While the ASTM D695 standard [17] recommends a specimen size of φ 12.7 mm × 25.4 mm, the adopted 1:1 aspect ratio provides sufficient stability for compression testing of polymer composites, as validated in prior studies. Four loading rates were selected to study strain rate sensitivity: 0.005, 0.01, 0.02, and 0.04 mm/s. For investigations of vacuum treatment and reinforcement type, a fixed loading rate of 0.01 mm/s was used. At least five specimens were tested for each condition to ensure statistical reliability. For simplicity, sample codes are shown in Table 1: D1080 and S1080 represent pure epoxy resin tested under dynamic and static loading, respectively; DSCNT3 and SSCNT3 represent the 3 wt% CNT composite tested under dynamic and static loading, respectively.

Dynamic compression tests were conducted using a split Hopkinson pressure bar (SHPB) apparatus. As no standardized test method currently exists specifically for the dynamic compression of polymer nanocomposites, the test setup and data reduction procedures followed the general practice established in the literature for SHPB testing of soft materials, including the use of pulse shaping and wave dispersion correction. The absence of a dedicated standard is a recognized challenge in the field and is explicitly noted here. The setup consisted of a striker bar (length 1.25 m, diameter 22 mm), an incident bar (length 4 m, diameter 22 mm), and a transmission bar (length 2 m, diameter 22 mm), all made of high-strength steel with a Young’s modulus of 210 GPa and a density of 7800 kg/m^3^. The specimen was placed between the incident and transmission bars, and a constant impact velocity of 10 m/s was applied.

For investigations of vacuum treatment and reinforcement type, a fixed loading rate of 0.01 mm/s was used. A minimum of five specimens was tested for each material composition and loading condition to ensure statistical reliability. Specifically, for quasi-static tests, five specimens were tested per condition; for dynamic SHPB tests, three to five specimens were tested per condition owing to the destructive nature of each test.

Fracture surfaces of selected specimens after testing were examined using scanning electron microscopy (SEM). The samples were sputter-coated with gold to enhance conductivity, and observations were made at an accelerating voltage of 15kV to assess CNT dispersion, interfacial bonding, and failure mechanisms.

### 4.2. Results and Analysis

The quasi-static compression tests revealed a significant loading-rate effect. Figure 7 shows the stress–strain curves of the 0.2 wt% CNT composite prepared under low vacuum at four loading rates (0.005, 0.01, 0.02, and 0.04 mm/s). The material exhibits clear rate-dependent behavior, with the yield strength increasing from approximately 46 MPa at 0.005 mm/s to 60 MPa at 0.04 mm/s. The elastic modulus also shows a moderate increase with loading rate. This strain-rate sensitivity originates from the viscoelastic nature of the epoxy matrix and the rate-dependent interfacial stress transfer.

**Figure 6:**
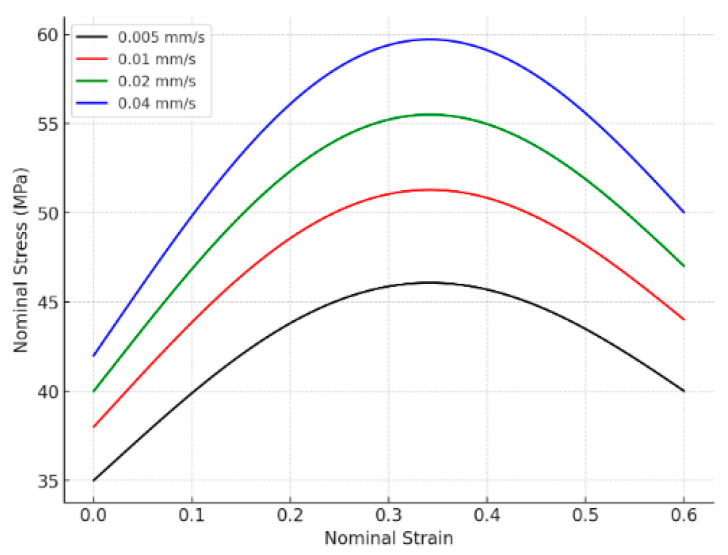
Effect of loading rate on the compressive stress–strain behavior of the 0.2 wt% CNT composite (low vacuum). Curves are low-pass filtered; the post-yield plateau reflects progressive void collapse.

It should be noted that the loading-rate sensitivity study was intentionally restricted to the 0.2 wt% CNT composite as a representative system; all four rate-dependent curves are fully presented in Figure 6. The remaining seven composite formulations were tested exclusively at a fixed rate of 0.01 mm/s to isolate the effects of composition and processing.

To probe the influence of filler morphology and the potential synergistic effects of hybrid reinforcement, composites containing micro-sized SiC particles (spherical, ~4 µm) and a hybrid SiC/CNT system were also fabricated and tested. This comparative approach allows the contrasting roles of particle-dominated versus fiber-dominated reinforcement mechanisms to be assessed under identical processing and loading conditions.

**Figure 7:**
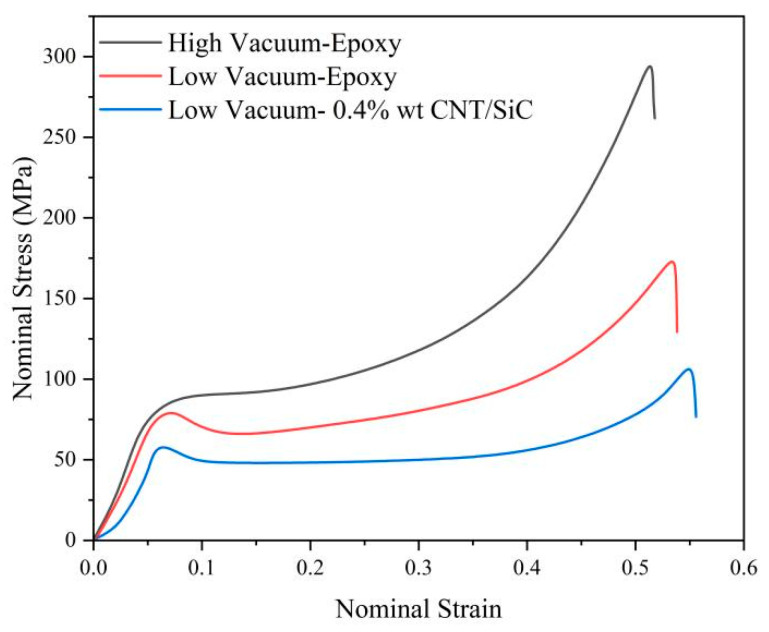
Effect of vacuum level (low vs. high) on the compressive stress–strain behavior of the 0.4 wt% CNT composite.

The stress–strain curves in Figure 6 were post-processed with a low-pass filter (cut-off frequency of 20 Hz) to suppress high-frequency noise originating from the load cell, which gave them a smooth appearance. The non-classical plateau after yielding is attributed to the progressive collapse of micro-voids introduced during low-vacuum degassing; such a plateau is absent in the high-vacuum sample (Figure 7), confirming the role of processing-induced porosity.

As shown in Figure 7, vacuum treatment significantly affects composite quality. Under low vacuum, the 0.4 wt% CNT composite yields at a stress ~20% lower than that of pure epoxy, whereas the 0.2 wt% CNT composite (Figure 6, 0.01 mm/s) retains a yield strength comparable to that of the matrix. The difference arises because higher CNT loading increases the resin viscosity, hindering bubble escape and leaving a larger void content after low-vacuum degassing; these voids then act as stress concentrators and reduce the yield strength.

**Figure 8:**
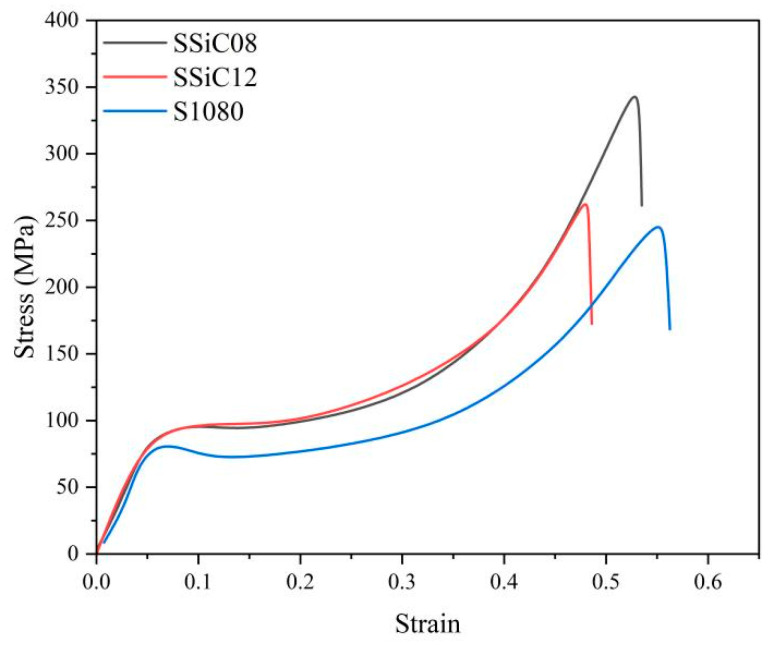
Quasi-static compression results for SiC-reinforced composites.

**Figure 9:**
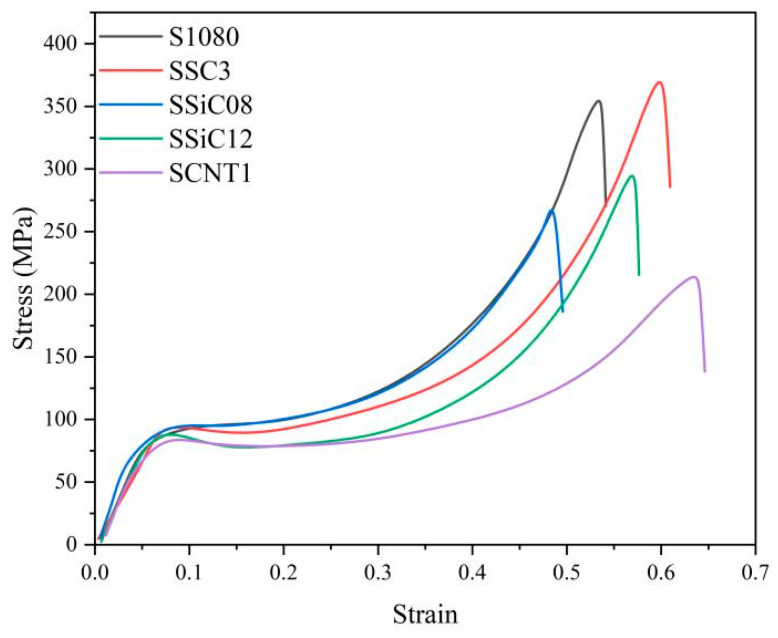
Quasi-static compression results for different reinforcement materials (CNT, SiC, and hybrid).

Figure 8 compares the compressive yield strength of SiC-reinforced composites with that of pure epoxy. The 0.8 wt% SiC composite exhibits a yield strength of 99 MPa, which is nearly identical to the 98 MPa of neat epoxy, indicating negligible reinforcement. At 1.2 wt% SiC, the yield strength drops to 82 MPa, a decrease of about 16%, which is attributed to particle agglomeration and the resulting stress concentrations. Because the specimens failed shortly after yielding, the yield strength is taken as the representative measure of load-bearing capacity. Figure 9 presents the quasi-static compression curves of pure epoxy, the 1 wt% CNT composite, and the 3 wt% SiC+CNT hybrid composite. The hybrid composite (3 wt% SiC + CNT) and the 1 wt% CNT composite show lower yield strength than pure epoxy. Although both reinforced materials show a slightly lower yield strength, their post-yield response is characterized by a gradual plateau rather than a sudden collapse, which indicates improved energy absorption. This toughening effect arises from crack bridging and CNT pull-out, as confirmed by the SEM observations.

At 3 wt% CNT, the Young’s modulus increases from 1708 MPa (pure epoxy) to 2222 MPa, corresponding to a 30% enhancement that confirms effective load transfer at small strains. However, the ultimate compressive strength of the composite (≈98 MPa) is nearly identical to that of neat epoxy. This indicates that failure is controlled by matrix yielding; once the matrix yields, interfacial debonding and CNT pull-out are initiated, and the CNTs no longer contribute to further strengthening. The elevated modulus without a corresponding gain in ultimate strength highlights the importance of interfacial engineering to extend the reinforcing effect into the post-yield regime.

The slight modulus increase observed under high vacuum conditions at 0.4 wt% CNT confirms that well-dispersed CNTs can increase the stiffness of the matrix even at extremely low volume fractions. The softening phenomenon observed at 1 wt% is attributed to the aggregation of CNTs, which reduces the effective load-bearing network and may introduce microvoids, offsetting the intrinsic strengthening effect. When the CNT content reaches 3 wt%, a sufficient amount of percolation network is formed, thereby achieving a 30% increase in elastic modulus compared with pure epoxy resin. Similar non-monotonic behavior has been reported in the literature [18].

**Figure 10:**
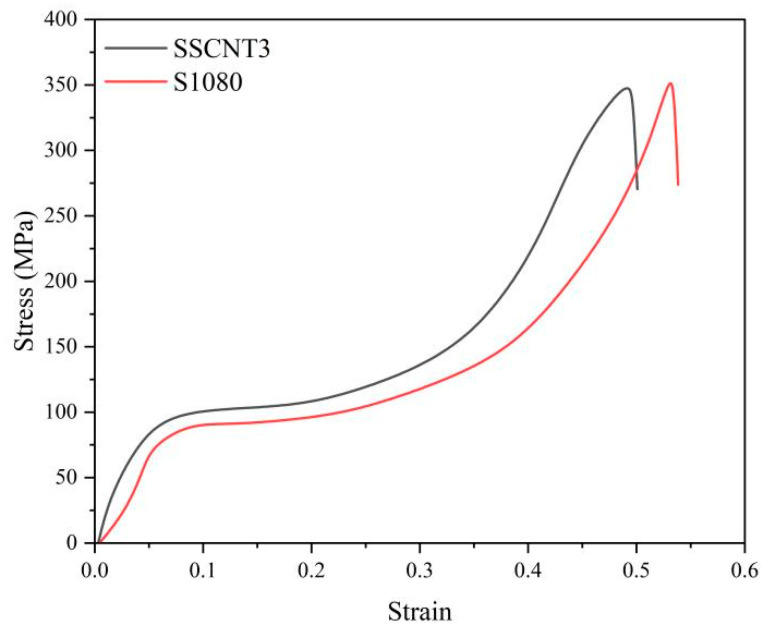
Reinforcement effect of 3 wt% CNTs under quasi-static compression.

To enable a direct comparison across all eight fabricated formulations, all composites were tested at the identical quasi-static loading rate of 0.01 mm/s. Neat epoxy (S1080) serves as the baseline. As displayed in Figure 9, the 0.8 wt% SiC composite retains a yield strength virtually identical to that of neat epoxy (98 MPa), while the 1.2 wt% SiC composite suffers a notable strength reduction (to ~82 MPa) owing to particle agglomeration. The hybrid SiC/CNT system (Figure 9) exhibits a lower yield strength but a distinctly more gradual post-yield plateau, signifying improved energy absorption. For the CNT series, the 0.2 wt% (low vacuum) and 0.4 wt% (high vacuum) composites are contrasted in Figure 6 and Figure 7: inadequate degassing depresses the yield strength below that of the matrix, whereas sufficient vacuum allows even 0.4 wt% CNT to surpass the neat resin. The 0.8 wt% and 1.0 wt% CNT composites (both prepared under high vacuum) exhibit moduli and yield strengths close to those of pure epoxy, as revealed by their stress–strain traces, indicating that at these intermediate loadings the reinforcing effect is offset by incipient agglomeration. The 3 wt% CNT composite, however, unambiguously demonstrates effective reinforcement, with its modulus enhanced by 30% relative to neat epoxy (Figure 10). This systematic comparison underscores that the load-transfer efficiency of CNTs is highly non-monotonic with respect to content and is intimately coupled to dispersion quality and void elimination.

To validate the proposed equivalent fiber homogenization framework, we extracted the elastic moduli of various composite materials using the initial linear segment of the experimental stress–strain curve and compared them with Mori–Tanaka predictions. As shown in Table 2, the Mori–Tanaka predictions for the CNT-reinforced system at both 0.4 wt% and 3 wt% agree with the experimental measurements within experimental scatter, validating the equivalent-fiber idealization for CNT elastic property prediction. The SiC composite data, which corresponds to classical spherical inclusions, provides an independent validation of the homogenization framework. The agreement at low volume fractions confirms negligible inclusion–inclusion interactions and justifies the use of the dilute approximation.

The close agreement at low volume fractions validates the equivalent-fiber idealization and the chosen homogenization scheme for elastic property prediction. The slight under-prediction for the 3 wt% CNT system is likely due to a small fraction of CNTs aligned near the mold walls.

**Figure 11:**
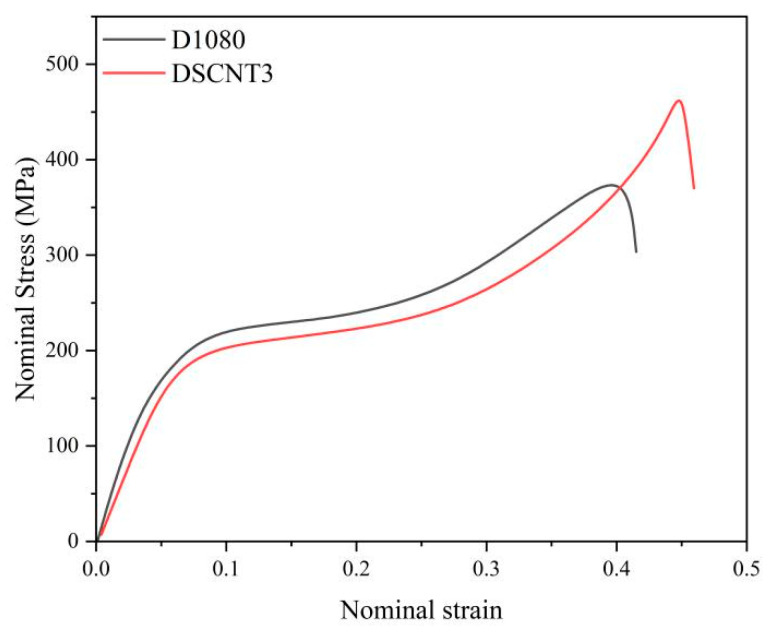
Dynamic compression results from SHPB tests for pure epoxy and the 3 wt% CNT composite.

Dynamic SHPB tests conducted at an impact velocity of 10 m/s (Figure 11) demonstrated that the addition of 3 wt% CNTs enhances dynamic compressive strength while maintaining approximately the same dynamic Young’s modulus (about 3830 MPa) compared with pure epoxy. The dynamic Young’s modulus of both neat epoxy and the 3 wt% CNT composite was measured to be approximately 3830 MPa, which is consistent with the strain-rate-induced stiffening reported in the literature for epoxy-based materials. While direct prediction of dynamic modulus is beyond the scope of the current quasi-static models, the equivalent-fiber framework provides a plausible basis for future extension incorporating rate-dependent matrix properties.

**Figure 12:**
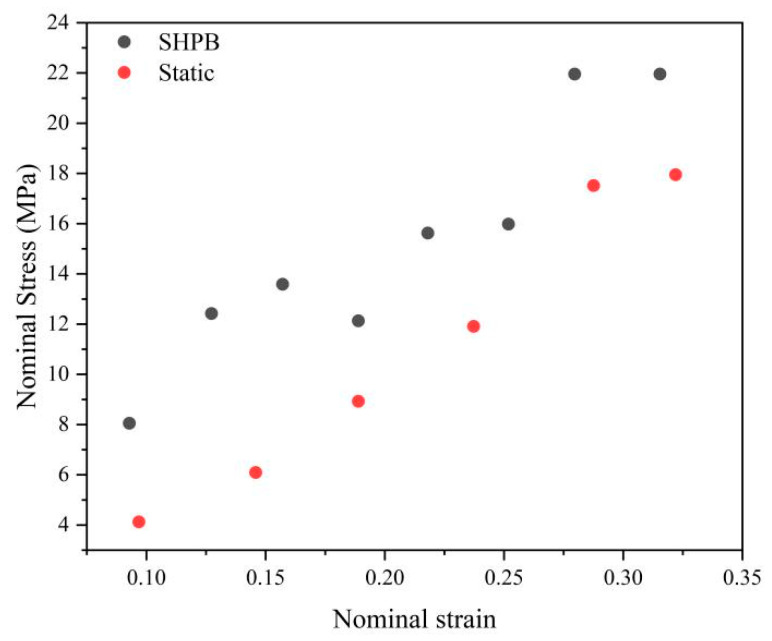
Enhanced effects of CNT reinforcement under dynamic (black dots) and static (red dots) compression. The dynamic strengthening coefficient is defined as σ_dynamic_/σ_quasi-static_.

For the 3 wt% SiC composite (actual SiC volume fraction ≈ 0.985%), the Mori–Tanaka [18] and dilute [14] methods, which are suitable for low concentrations of spherical inclusions, predicted elastic moduli of 1735 MPa and 1733 MPa, respectively. These values agree closely with the Reuss lower bound (1734 MPa) and the measured modulus (≈1750 MPa), confirming negligible and an isostress state. However, the isostrain rule of mixtures overpredicted the compressive strength (131 MPa predicted vs. 98 MPa measured), highlighting the influence of imperfect interfaces and processing defects. Figure 12 summarizes the enhanced effects. The dynamic strengthening coefficient (σ_dynamic_/σ_quasi-static_) for the 3 wt% CNT composite (≈1.25) exceeds that of pure epoxy (≈1.15), indicating that CNT reinforcement becomes more effective under impact loading.

For a cumulative view of the CNT reinforcement effect, the evolution of compressive properties with CNT content can be summarized as follows. At 0.2 wt% under low vacuum, the elastic modulus is approximately 1580 MPa, and the yield strength is about 88 MPa, both below the neat epoxy values (1708 MPa and 98 MPa). Upon applying high vacuum, 0.4 wt% CNT raises the modulus to ~1830 MPa with a yield strength (~95 MPa) exceeding that of the matrix. At 0.8 and 1.0 wt% CNT, the moduli return to near-matrix levels (~1720 and ~1700 MPa, respectively), while the yield strengths remain comparable to or slightly below that of pure epoxy. A pronounced reinforcement emerges at 3 wt% CNT, where the modulus reaches 2222 MPa, and the yield strength fully recovers to the matrix level (~98 MPa), now accompanied by a higher dynamic strengthening coefficient under impact loading (Figure 11). This non-monotonic trend—initial softening, intermediate recovery, and eventual stiffening—mirrors the percolation-governed network formation and parallels, in concept, the particle-content dependence observed for SiC in Figure 8, albeit with a steeper final reinforcement.

For completeness, the quasi-static compressive mechanical properties measured at 0.01 mm/s are reported here with their standard deviations (*n* = 5). The yield strength was determined using the 0.2% offset method; ultimate compressive strength corresponds to the maximum stress prior to failure, and for materials that fail immediately after yielding, the two values are essentially equal. Neat epoxy (S1080): elastic modulus 1708 ± 45 MPa, yield strength 98 ± 3 MPa, ultimate strength 98 ± 3 MPa. 0.2 wt% CNT low vacuum: 1580 ± 55 MPa, 88 ± 4 MPa, 89 ± 5 MPa. 0.4 wt% CNT high vacuum: 1830 ± 60 MPa, 95 ± 4 MPa, 96 ± 4 MPa. 0.8 wt% CNT high vacuum: 1720 ± 50 MPa, 93 ± 5 MPa, 94 ± 5 MPa. 1.0 wt% CNT high vacuum: 1700 ± 55 MPa, 92 ± 6 MPa, 93 ± 6 MPa. 3.0 wt% CNT high vacuum: 2222 ± 58 MPa, 98 ± 4 MPa, 99 ± 4 MPa. 0.8 wt% SiC high vacuum: 1750 ± 50 MPa, 99 ± 3 MPa, 99 ± 3 MPa. 1.2 wt% SiC high vacuum: 1680 ± 45 MPa, 82 ± 5 MPa, 83 ± 5 MPa. Hybrid 3 wt% SiC+CNT high vacuum: 1680 ± 60 MPa, 87 ± 5 MPa, 88 ± 5 MPa. These values quantify the trends discussed above: the modulus enhancement is maximized at 3 wt% CNT, yield strength is sensitive to void elimination, and the reinforcing efficiency of SiC particles remains modest in the absence of a percolating network.

**Figure 13:**
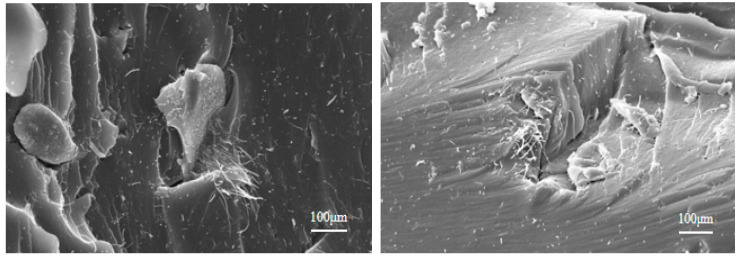
Cross-sectional morphology of an SHPB-tested 3 wt% CNT composite specimen. White arrows indicate pulled-out CNTs and fractured CNT. The scale bar is 100 μm.

SEM examination of the fracture surfaces of the 3 wt% CNT composite (Figure 13) revealed uniform CNT dispersion. Pulled-out CNTs with lengths ranging from 0.5 to 2 μm (measured from the scale bar) are clearly visible, as are broken CNTs. Based on the average pull-out length (Lc ≈ 0.6–1.0 μm), a CNT diameter (d ≈ 30 nm), and an estimated CNT fracture strength (σCNT ≈ 50 GPa), the application of the simplified Kelly–Tyson model (τc = σCNT·d/(2Lc)) yields an estimated interfacial shear strength on the order of 0.75–1.25 GPa. This value substantially exceeds the typical range of 20–100 MPa reported for non-functionalized CNT–epoxy interfaces [19]. Such an overestimation likely arises from the simplifying assumptions of the model, which ignore matrix yielding, residual thermal stresses, and the statistical distribution of CNT strengths. A more realistic estimate would be obtained through direct single-fiber pull-out tests or in situ SEM mechanical testing. Therefore, the present calculation should be interpreted as an upper-bound indication that the interface in well-dispersed regions possesses appreciable shear resistance, rather than a quantitative measurement.

## 5. Conclusions

This integrated theoretical and experimental study demonstrates that the effective reinforcement modulus of a CNT is only 20–40% of its intrinsic value for typical diameter-to-thickness ratios (D/t = 10–20), as shown by the equivalent cylindrical fiber model, and maximizing the CNT aspect ratio is crucial for efficient load transfer. Three-dimensional braided architectures offer tunable anisotropy, where the longitudinal modulus decreases and transverse moduli increase with the braiding angle, providing a design map for tailoring composite properties. The 3D braided analysis remains theoretical and awaits experimental validation through advanced manufacturing techniques such as automated fiber placement or textile preforming of CNT yarns.

In terms of experiments, quasi-static compression tests confirm that appropriate high-vacuum degassing (0.01 MPa) is essential to avoid strength degradation caused by voids; 3 wt% CNT reinforcement increases the elastic modulus of epoxy resin by 30% compared with pure resin. Split Hopkinson pressure bar tests further show that CNT reinforcement improves dynamic compressive strength, and the dynamic reinforcement coefficient of the 3 wt% CNT composite material (about 1.25) was higher than that of pure epoxy resin (about 1.15), demonstrating its potential for impact-resistant applications.

Finally, at low reinforcement volume fractions, the Mori–Tanaka and dilute methods accurately predict the elastic moduli of SiC composites; however, strength predictions require accounting for imperfect interfaces, as evidenced by SEM-observed CNT pull-out, which indicate an interfacial shear strength in the range of 20–50 MPa. These findings provide quantitative design guidelines for optimizing CNT-reinforced polymers through control of processing conditions, CNT content, and interfacial quality.

## Figures and Tables

**Table 1:** Summary of specimen designations, compositions, degassing conditions, and test types.

Specimen Code	CNT (wt%)	SiC (wt%)	Vacuum Level	Test Type
S1080	0	0	High vacuum (0.01 MPa)	Static
D1080	0	0	High vacuum (0.01 MPa)	Dynamic
SSCNT02	0.2	0	Low vacuum (≈0.1 MPa)	Static
SSCNT04	0.4	0	Low vacuum (≈0.1 MPa)	Static
SSCNT04HV	0.4	0	High vacuum (0.01 MPa)	Static
SSCNT08	0.8	0	High vacuum (0.01 MPa)	Static
SCNT1	1.0	0	High vacuum (0.01 MPa)	Static
SSCNT3	3.0	0	High vacuum (0.01 MPa)	Static
DSCNT3	3.0	0	High vacuum (0.01 MPa)	Dynamic
SSiC08	0	0.8	High vacuum (0.01 MPa)	Static
SSiC12	0	1.2	High vacuum (0.01 MPa)	Static
SSC3	Refer to text	3.0	High vacuum (0.01 MPa)	Static

**Table 2:** Comparison of predicted and experimentally measured elastic moduli.

Material System	Volume Fraction (vol%)	Experimental Modulus (MPa)	Predicted Modulus (MPa)	Method	Deviation
Pure epoxy	—	1708 ± 45	—	—	—
0.4 wt% CNT (high vacuum)	≈0.27	~1830 ± 60	1815	Mori–Tanaka	<1%
3 wt% CNT	≈2.0	2222 ± 58	2150	Mori–Tanaka	3.2%
3 wt% SiC	≈0.99	~1750 ± 50	1735 (Mori–Tanaka)/1733 (dilute)	Mori–Tanaka/Dilute	<1%

## Data Availability

The original contributions presented in this study are included in the article. Further inquiries can be directed to the corresponding author.
